# Durum wheat miRNAs in response to nitrogen starvation at the grain filling stage

**DOI:** 10.1371/journal.pone.0183253

**Published:** 2017-08-16

**Authors:** Diana L. Zuluaga, Domenico De Paola, Michela Janni, Pasquale Luca Curci, Gabriella Sonnante

**Affiliations:** 1 Institute of Biosciences and Bioresources, National Research Council, Bari, Italy; 2 Institute for Electronics and Magnetism, National Research Council (CNR), Parma, Italy; Dokuz Eylul Universitesi, TURKEY

## Abstract

Durum wheat highly depends on nitrogen for seed development and yield, and the obtainment of varieties with a better nitrogen use efficiency is crucial to reduce production costs and environmental pollution. In this study, sequencing of two small RNA libraries obtained from tissues of Ciccio and Svevo cultivars grown under nitrogen starvation conditions produced 84 novel, and 161 conserved miRNAs. Of these, 7 novel and 13 known miRNAs were newly identified in this work. Quantitative PCR analysis of selected miRNAs highlighted that the expression levels of some of them depends on the tissue and on the cultivar, Svevo being the most responsive to nitrogen starvation. A number of target genes were predicted to be involved in nitrogen metabolism. An inverse correlation for the qPCR expression data of miRNA/target pairs miR399b/*PHO2*, miR393c/*AFB2*, ttu-novel-61/*CCAAT-TF* was observed in specific tissues or cultivar. Especially, ttu-novel-61 was down-regulated and its target *CCAAT-TF* up-regulated in almost all tissues both in Svevo and in Ciccio. Moreover, *CCAAT-TF* was confirmed to be cleaved by ttu-novel-61 at the expected site. The discovery of miRNAs involved in the response to nitrogen stress represents an important step towards functional analyses, with the final aim to design strategies for improving nitrogen use efficiency in durum wheat.

## Introduction

The allotetraploid durum wheat [*Triticum turgidum* subsp. *durum* (Desf.) Husn.] is among the most important cereal crops especially in the Mediterranean basin where it is used for the preparation of pasta and many other typical products [1]. The high economic importance of durum wheat and the recent availability of the hexaploid bread wheat (*T*. *aestivum* L.) draft genome has sped up the studies of genomics and transcriptomics on wheat species. However, the miRNomics of durum wheat are still at the beginning and a lot is yet to be discovered [2,3].

Abiotic stress negatively affects the adaptability to the environment and the productivity of cereal crops worldwide [4]. Crop productivity largely depends on the availability and use of nitrogen (N) due to its essential role as a macronutrient for plant growth and development [5,6].

Nitrogen (N) use by plants includes several steps, namely: uptake, assimilation, translocation, recycling and remobilization [7]. The metabolic pathways involved in these processes have been well described in *Arabidopsis thaliana* [6,7] as well as in some crop plants [8]. In cereals, during the grain filling period, N uptake and assimilation is often insufficient for the high demand from the seeds, so several remobilization steps occur in the plant organs and in particular, proteins from the flag leaf are extensively degraded, providing a great source of N needed for seed development [7].

When N is not available in the wheat growing environment, grain dry matter yield and N content decrease [5]. Therefore, in order to improve yield in these conditions, the use of N fertilizers has dramatically increased over the past decades. The study of the transcriptome response to N starvation in rice, showed that a total of 3518 genes, representing almost 11% of the rice genome, were responsive to N starvation by altering their transcript levels [9]. On the other hand, it is important to explore the physiological and molecular variations occurring in plant germplasm in response to nitrogen availability. In fact, the transcriptomic response of two maize genotypes with a different NUE to NO3(-) induction was completely different [10]. A crucial and sustainable strategy to minimize the application of chemicals in N starvation conditions is the improvement of N use efficiency in crops through the regulation of key genes involved in N metabolism pathways.

MicroRNAs (miRNAs) are small non-coding RNAs that play important roles in the modulation of gene expression [11–14]. Several studies demonstrate the critical role of miRNAs in the regulation of plant responses to biotic and abiotic stresses, which are vital determinants in the production of cereals [15]. The understanding of the molecular mechanisms underlying abiotic stress responses, including gene regulation through miRNAs, represents the starting point for the development of stress-tolerant cereal varieties [16,17]. Within the Poaceae family, several rice and maize miRNAs have been described to be involved in responses to nitrogen deficiency. Analysis of the dynamics of rice transcriptome under N starvation suggests a potential role in plant nutrient homeostasis of miR399 and miR530 [9]. Moreover, nine miRNAs were found differentially expressed in rice under low-N conditions: miR156, miR164, miR528, miR820, miR821 and miR1318 in leaves and miR164, miR167, miR168 and miR528 in roots [16]. In maize, a small RNA analysis identified eight miRNA families differentially expressed under N-deficient condition, including miR169, miR395, miR528, and miR827 family members [18]. Another study [19] provided an in-depth analysis of miRNAs and degradome in maize seedlings, showing that two miR169 family members play an important role in the adaptation of maize to low N conditions.

Some recent studies have identified nitrogen responsive miRNAs in bread wheat. For instance, Sinha and co-workers [20] showed notable differences in expression pattern of miR159a, miR159b, miR399, and miR408 under low-N conditions (0.04mM) as compared to 4mM nitrate supply in root tissue of two highly N-responsive bread wheat genotypes. A recent study has identified differential expression levels of tamiR156, tamiR399, tamiR444, tamiR1118, tamiR1129, tamiR1133, tamiR1136 in root tissue of bread wheat under N deprivation compared to normal conditions [21]. Furthermore, tamiR444a, has been well characterized and its role in mediating plant tolerance to N-starvation stress has been confirmed [22]. Based on microarray and qPCR analyses, it has been shown that the altered transcription of nitrate transporter (NRT) and antioxidant enzyme-encoding (AEE) genes is associated with the improvement of the N acquisition and cellular ROS (reactive oxygen species) detoxification in tobacco tamiR444a-overexpressing plants [22].

A number of miRNAs in durum wheat leaves and roots have been identified, some of which are related to development processes [3]. Other durum wheat miRNAs regulate water-deficit stress responses [2]. For instance, Liu and colleagues identified 66 conserved and five novel miRNA differentially abundant under water deficit stress [2] and validated nine mRNA targets cleaved by water-deficit stress-responsive miRNAs [23]. Additionally, the same group suggested a pre-anthesis water-deficit stress responsive mechanism regulated by miR160-*ARFs* in durum wheat flag leaf and head [24]. However, so far, there is no information about the miRNAome involved in durum wheat tolerance to N deficiency stress.

In this work, we provide an insight into durum wheat conserved and novel miRNAs and their target genes regulating the adaptation processes to nitrogen stress during the grain filling developmental stage. To this aim, two sRNA libraries were obtained from tissues of Ciccio and Svevo cultivars grown under N deficiency. Libraries were sequenced, and selected miRNAs and target genes were validated through qPCR and, in one case, also by means of 5’RACE assay.

## Materials and methods

### Plant material and phenotypic analyses

Seeds of the durum wheat cultivars Ciccio and Svevo were sterilized as in De Paola et al. [3] and then kept for 10 days at 4°C. Plantlets were transferred into 10 cm diameter net pots containing 80% agriperlite and 20% expanded clay, and grown in a hydroponic system under N stress, in a greenhouse at 18–20°C under day light photo-period. Hydroponic solution was prepared as reported in Curci et al. [25], growing plants under N stress condition, with 0mM Ca(NO_3_)_2_*4H_2_O. Roots were submerged all the time in the solution, which was continuously aerated and refreshed every two days. This set of plants was grown simultaneously with the durum wheat plants raised under standard N conditions [3].

At the same Z77 developmental stage, roots, leaves, flag leaf, and spikes were collected from three individual plants from Ciccio and Svevo separately. Plant material was immediately frozen at -80°C.

For phenotypic analysis, the following traits were scored: plant height (PH), flag leaf area (FLA), number of culms per plant (NCPP), number of spikelets per spike (NSPS), spike dry matter (SDM), flag leaf dry matter (FLDM), kernel number per spike (KNPS), kernel weight per spike (KWPS).

### Small RNA library preparation

For small RNA (sRNA) isolation, mirPremier microRNA Isolation Kit (Sigma-Aldrich, St. Louis, MO, USA) was used with 100 mg of each tissue from each plant. For Ciccio and Svevo separately, a pool was obtained by mixing an equal amount of sRNA from the four tissues of the three plants.

Two sRNA libraries from Ciccio and Svevo N stressed tissues were prepared using TruSeq Small RNA Sample Preparation (Illumina, San Diego, USA). Sequencing was performed at the Institute of Applied Genomics (IGA, Udine, Italy).

### Detection of conserved and novel miRNA

Sequencing data for miRNA detection were treated as in De Paola et al. [3]. Briefly, low quality sequences and adapters were removed and unique RNAs were counted. Each count was normalized and expressed as transcript per million (TPM). Unique reads were blasted for similarity against plant mature miRNAs in miRBase (release 21), with the aim to identify conserved miRNAs considering only sequences with no mismatch. Blastn was run locally by command line with the task option blastn-short. Durum wheat miRNAs already identified in De Paola et al. [3] were also searched in the durum N stressed libraries. For novel miRNA identification, a custom pipeline was used, and precursors were detected by blasting novel miRNA sequences against durum wheat ESTs and *T*. *aestivum* unigenes in TAGI database version 12 (ftp://occams.dfci.harvard.edu/pub/bio/tgi/data/Triticum_aestivum/TAGI.release_12.zip). MirRNA secondary structure was predicted by folding sequences by means of UNAfold [26]. Identification of conserved and novel miRNAs was performed following the criteria of Meyers et al. [27], and for all miRNAs, sequences with at least 5 counts in one of the four libraries (including control and N stressed libraries) were taken into account.

### MicroRNA expression

For the quantitative PCR (qPCR) validation of selected miRNAs, sRNA fraction was extracted using the mirPremier microRNA Isolation Kit (Sigma-Aldrich, USA), and its quality was checked by electrophoresis in 1% agarose gel containing 1% gel red (Biotium, Hayward, CA, USA). Analyses were carried out as described in Varkonyi-Gasic et al. [28], using the Universal Probe Library (UPL) assay. Each reaction contained 5 μl of 2x Rotor-gene Probe PCR Master Mix (Qiagen, Hilden, Germany), 0.5 μl of miRNA-specific forward primer (10 μM), 0.5 μl of the universal reverse primer (10 μM) (S1 Table), 0.1 μl of Universal Probe Library, Probe #21 (Roche Diagnostics GmbH, Mannheim, Germany), 3.4 μl of nuclease-free water and 0.5 μl of the respective retro-transcribed miRNA. Primers for expression analysis were designed based on miRNA sequences. PCR reactions were performed in a Rotor-Gene 6000 machine (Corbett Life Science, Sydney, Australia), with the following conditions: an initial step at 95°C for 5 min, followed by 40 cycles of 95°C for 5 sec, 60°C for 10 sec and 72°C for 1 sec. Each experiment included three technical replications of three different plants and a no-template control, and was performed on sRNA from control plants and plants grown under N stress. For the choice of housekeeping miRNA, a preliminary qPCR analysis was carried out on N stressed and control plants to evaluate four miRNAs, which were not reported to be affected by nitrogen stress in other plant species: ttu-miR171, ttu-miR1130-b, ttu-miR168. MiRNA expression stability for normalization was determined by means of GeNorme software.

Relative expression of miRNAs was analyzed using the comparative C_T_ method [29]. MiRNAs showing > 2 fold change between control and N stressed plants were considered as differentially expressed. According to independent Student’s *t* test analysis, fold changes with a p-value < 0.05 were considered statistically significant.

### Prediction and validation of target genes

The web tool psRNATarget (http://plantgrn.noble.org/psRNATarget/) [30], was used to detect potential target genes for miRNAs of the N-stressed sRNA libraries. Three transcript libraries were employed for target search: durum wheat ESTs (NCBI database), *T*. *aestivum* unigenes from DFCI Gene Index (TAGI) version 12, and *Hordeum vulgare* unigenes from DFCI Gene Index (HVGI), version 12. All targets obtained and their membership were analyzed using Venny 2.1 (http://bioinfogp.cnb.csic.es/tools/venny/). The sequence of the target gene *PHO2* was retrieved from proprietary durum wheat RNAseq sequences available in our laboratory.

For target gene expression, total RNA was isolated from Ciccio and Svevo N-stressed and control plants using the RNeasy Mini Kit (Qiagen) and cDNA was reverse-transcribed by means of the Script cDNA Synthesis Kit (Bio-Rad Laboratories, Richmond, CA, USA) following the manufacturer’s instructions. Preliminary standard PCR and sequencing analyses were performed to verify primer quality, see S1 Table and to confirm the specificity of target gene fragments including the miRNA binding site. Real-time qPCR experiments were carried out in a 3500 Genetic Analyzer (Thermo Fisher, Rodano MI, Italy) using 20 ng of cDNA and the iQ SYBR Green Supermix (Bio-Rad Laboratories, Richmond, CA, USA) following the manufacturer’s protocol. Amplification conditions were: 95°C for 3 min, 40 cycles of 95°C for 15 sec and 60°C for 30 sec, followed by a melt curve profile. The reference gene was chosen by testing three genes encoding for the “ADP-RF(a) Ta2291 ADP-ribosylation factor”, the “RLI(a) Ta2776” similar to *A*. *thaliana* RNase L inhibitor protein (RLI(a)) and the cell division control protein, AAA-superfamily of ATPases [31]. PCR expression analysis was carried out on Svevo N-stressed and non-stressed tissues. Data for the normalizing gene were analyzed using GeNorme software. The relative quantification of each gene was done using the comparative C_T_ method [29], with RLI(a) as the reference gene. Putative target genes were considered as differentially expressed when showing > 2 fold change between N stressed and non-stressed conditions. Fold changes with a Student’s *t* test p-value < 0.05 were deemed statistically significant.

Target gene validation through 5’ RACE assay [32] was performed using the 5’ RACE System for Rapid Amplification of cDNA Ends (Thermo Fisher/Invitrogen) on total RNA from Svevo stressed roots, following the manufacturer instructions. Gene-specific reverse primers for 5’ RACE were designed on the basis of putative target sequence (S1 Table). PCR product was cloned and sequenced to confirm cleavage at the expected site.

## Results

### Phenotype analyses

In order to assess whether N stress conditions caused a different effect on the two durum wheat cultivars used in the present work, phenotypic traits were determined on Ciccio plant parts and compared to the phenotypic analysis of Svevo cultivar, which was previously used for a transcriptomic study [25]. Ciccio plants showed a severe growth depletion in all organs/tissues of plants grown under N deficiency (see S2 Table). Relative reduction (RR) ranged from lower values for PH (34.57%) and NSPS (39.48%), to higher levels in all the other plant organs (RR from 73.33 to 91.15 for NCPP and FLDM, respectively). The relative reduction for traits in stressed plants was higher in Ciccio than in Svevo [25], except for NCPP, where the values for the two cultivars were comparable.

### Detection of conserved and novel miRNAs in N stressed libraries

In order to identify durum wheat miRNAs involved in the regulation of N metabolism during the grain filling stage, two small RNA libraries were generated and sequenced from a mix of leaves, flag leaves, roots and spikes of Ciccio (SRA-NCBI accession SRP069277) and Svevo (SRA-NCBI accession SRP069277), separately. Plants were grown in conditions of N deprivation and tissues were collected at the stage of late milk (Z77). Sequencing data were filtered to remove low quality reads and adaptors, and subsequently, total reads amounted to 15,620,564 and 14,607,724 for Ciccio and Svevo, respectively. The RNA sequences belonging to snoRNAs, snRNAs, tRNAs, and rRNAs were filtered out by searching against ncRNAs in Rfam (https://www.sanger.ac.uk/).

Conserved miRNAs in durum wheat N stressed libraries were identified by mapping all the unique reads comprised between 18 and 31 nucleotides to known plant miRNAs in miRBase release 21. Considering a minimum read count equal to 5, a total of 161 known miRNAs were found in the two stressed libraries, 148 in Ciccio and 144 in Svevo. Most of the conserved miRNAs detected in Svevo plants under N starvation were in common with previously identified miRNAs in De Paola et al.[3], while thirteen of them were newly found in this work: ttu-miR167k, ttu-miR171f, ttu-miR172, ttu-miR319g, ttu-miR393d, ttu-miR395, ttu-miR396j, ttu-miR397a, ttu-miR399c, ttu-miR1122, ttu-miR2275, ttu-miR9654a, ttu-miR9677 (see S3 Table).

Moreover, seven additional putative novel miRNAs were predicted in this work with at least five counts in one of the libraries, three in Svevo and five in Ciccio (see S4 Table), which were not detected or were counted less than five in the control libraries [3].

**Fig 1:**
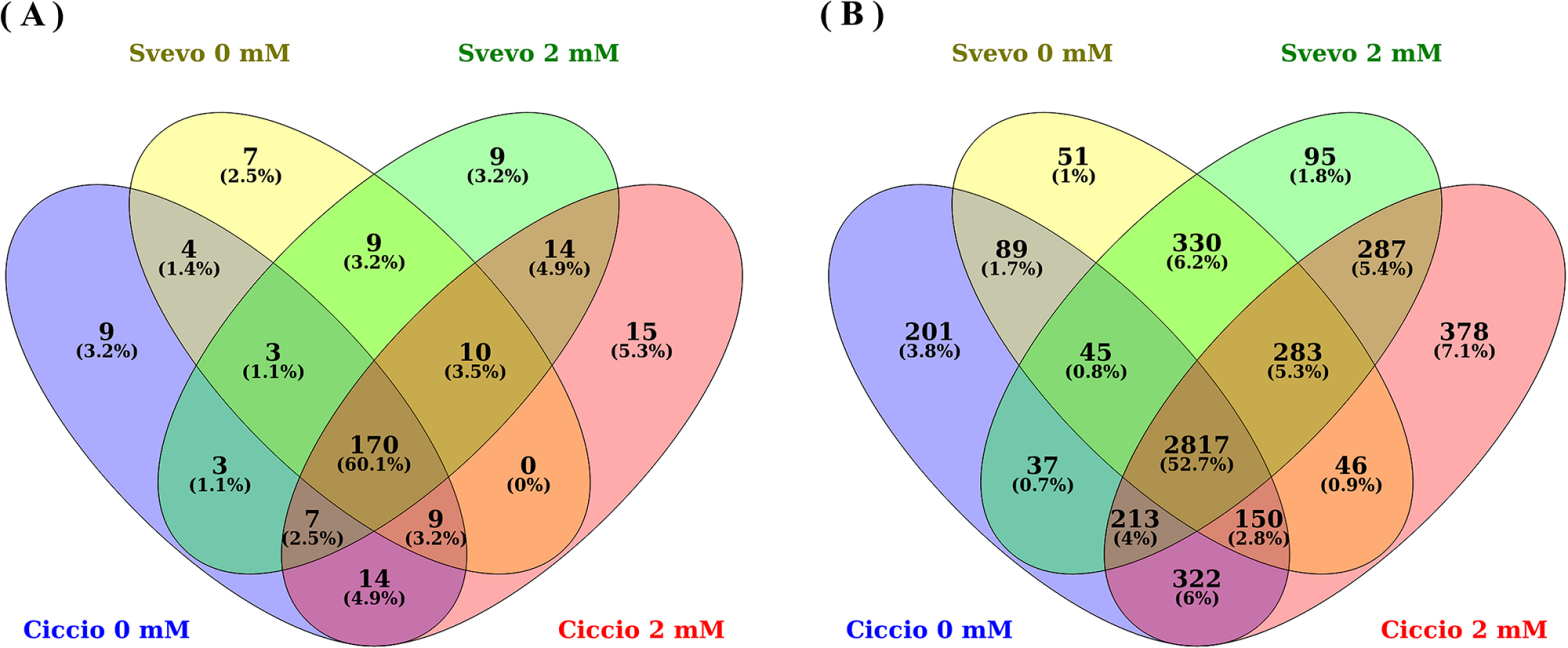
Venn diagram of durum wheat miRNAs and target genes identified in nitrogen deprivation conditions. Conserved and novel durum wheat miRNAs (A) and target genes (B) identified in four small RNA libraries, two from Ciccio and Svevo cultivars grown in nitrogen deprivation conditions (0mM nitrogen, this work), and two from the same cultivars grown in standard conditions (2mM nitrogen, De Paola et al., 2016); the number of miRNAs (A) or target genes (B) present in one or more libraries is indicated.

A comparison was performed among the miRNAs obtained from the four single sRNA libraries, two from Ciccio and Svevo grown under N deprivation conditions (this work), and two from the same varieties cultivated under standard conditions [3]. MiRNAs with at least five counts were considered for each library. Out of a total of 283 non-redundant miRNAs, 170 were common for the two cultivars in both the experimental conditions. Thirty-eight and 25 miRNAs were present only in Ciccio or in Svevo, respectively, while 20 miRNAs were found only in N stressed libraries (Fig 1A).

### Expression analysis of selected microRNAs

Several of the conserved miRNAs found in durum wheat small RNA libraries are reported to be regulated by nitrogen availability in plant species. In order to unravel the possible involvement of these miRNAs in durum N stress, we selected 14 (nine conserved and five novel) miRNAs, mainly chosen on the basis of their predicted target. The relative expression was analyzed by qPCR at the late milk developmental stage (Z77) [33], in roots, leaves-stems, flag leaf and spikes of Svevo and Ciccio, both in control (2mM N) and in stressed plants (0mM N).

To choose a suitable reference gene, three miRNAs (ttu-miR171, ttu-miR1130-b, ttu-miR168) were selected and evaluated as putative normalizers in our experiment, since they were not described as involved in nitrogen stress in plants. The qPCR expression profiles were analyzed for miRNA stability, and ttu-miR168 was chosen as normalizer since it proved to be the most stable miRNA in our experimental conditions.

**Fig 2:**
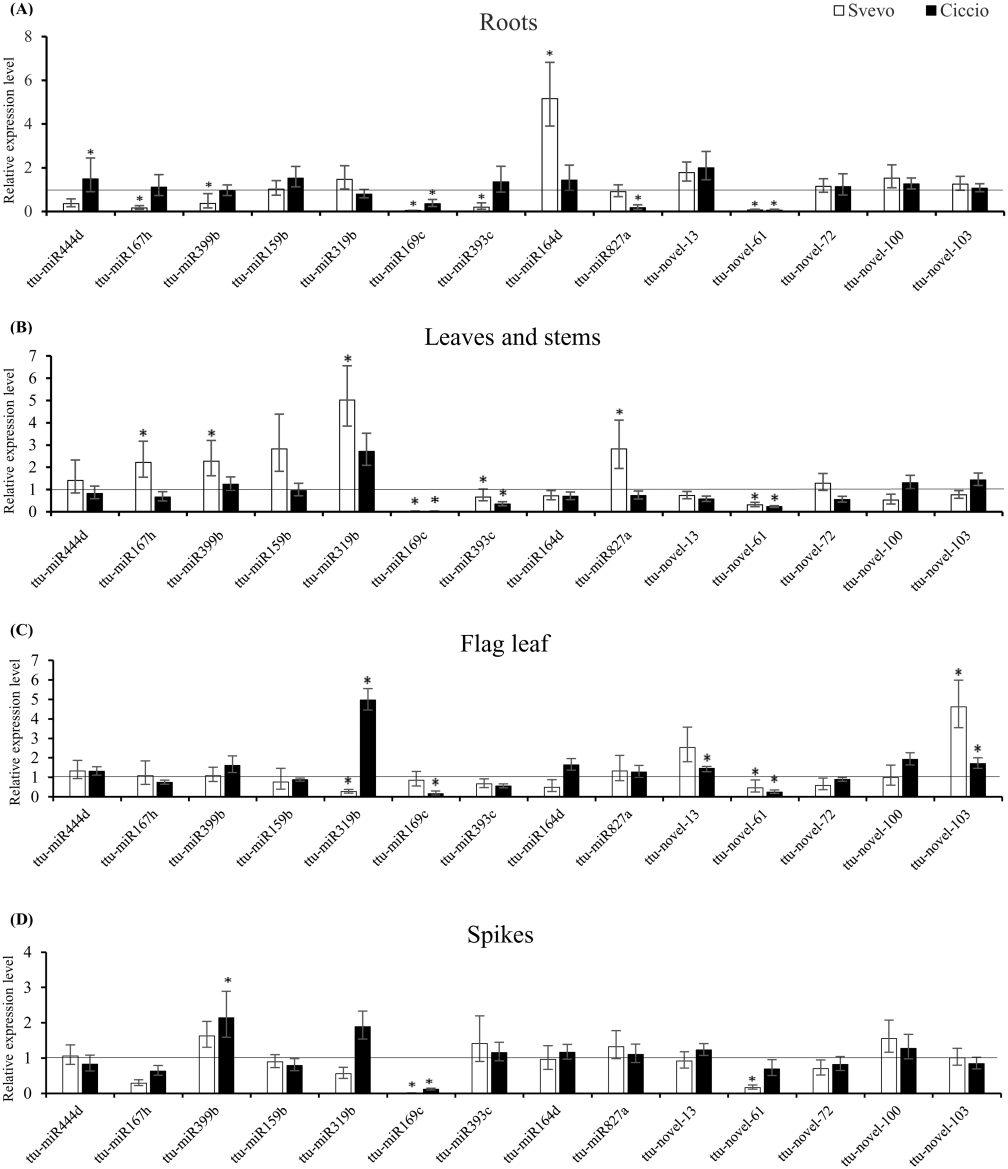
Expression analysis of selected miRNAs in durum wheat at the late milk stage (Z77). Selected conserved and novel miRNAs in roots (A), leaves/stems (B), flag leaf (C), and spikes (D) of Svevo (white bars) and Ciccio (black bars). Relative expression levels are given as fold change of the expression in nitrogen (N) stress conditions (0mM N) versus standard conditions (2mM N). Standard condition is arbitrarily set to 1 and indicated with a black line. Data are means ±SE. *P* values between three N stressed biological replicates and three replicates grown under standard N conditions, were calculated using unpaired, 2-tailed student’s *t* test for each miRNA. Asterisks indicate statistically significant values (p-value < 0.05).

Several conserved miRNAs selected for qPCR were found differentially expressed between N stressed and non-stressed tissues from Ciccio and Svevo plants. In Svevo roots, ttu-miR167h, ttu-miR169c, ttu-miR393c, and ttu-miR399b were significantly down-regulated compared to the control, with a fold change ranging from 0.040 (ttu-miR169c) to 0.369 (ttu-miR399b). Conversely, in the same tissue, ttu-miR164d was strongly up-regulated (5.162 times) under N stress. In Ciccio roots, ttu-miR169c and ttu-miR827a were down-regulated (0.356 and 0.171 times, respectively), while ttu-miR444d was slightly up-regulated in N stress conditions (Fig 2A). As in roots, in Svevo leaves and stems, ttu-miR169c and ttu-miR393c appeared down-regulated (0.020 and 0.669 times, respectively), while ttu-miR167h, ttu-miR399b, ttu-miR319b, and miR827a were up-regulated in N starvation conditions, with a fold change above 2, especially high for ttu-miR319b (5.027). For Ciccio leaves and stems, again ttu-miR169c and ttu-miR393c were strongly down-regulated (Fig 2B). In flag leaf and spike, fewer conserved miRNAs were differentially expressed in comparison with the other two tissues. MiR319b was the only conserved miRNA differentially expressed in Svevo flag leaf, being down-regulated compared to the control. In Ciccio flag leaf, once again, ttu-miR169c was strongly down-regulated, while miR319b was highly up-regulated in N stress environment (Fig 2C). In the spikes, ttu-miR169c was strongly down-regulated both in Svevo and in Ciccio, while ttu-miR399b was the only conserved miRNA up-regulated in Ciccio in this tissue (Fig 2D).

Five miRNAs (ttu-novel-13, ttu-novel-61, ttu-novel-72, ttu-novel-100, and ttu-novel-103), newly identified in this work or in De Paola et al. [3], were considered interesting for analysis by qPCR since their putative target genes, identified *in silico*, encode for enzymes playing a role in N metabolism. Three of these miRNAs (ttu-novel-61, ttu-novel-72, and ttu-novel-100) showed a read count lower than 5 in the stressed libraries. Our data showed that ttu-novel-61 was strongly down-regulated in N stress conditions in all tissues of Svevo and three Ciccio tissues (leaves/stems, flag leaf, and roots), with very low expression values in N stressed roots in both cultivars (below 0.07 fold change, Fig 2). In flag leaf from both cultivars, ttu-novel-103 was up-regulated in 0mM N compared to 2mM N plants, particularly in Svevo (fold change above 4.6). In Ciccio, ttu-novel-13 was also up-regulated in flag leaf under N stress conditions (Fig 2C).

### Target gene prediction and analysis

Target genes for the ttu-miRNAs, detected in the sRNA libraries and with at least five counts, were predicted using durum wheat ESTs, plus bread wheat and barley unigenes. Unique target gene accession numbers were considered, however, redundancies may still be observed due to the presence of multiple accession numbers for the same gene in the sequence databases. Target genes for the miRNAs newly detected in this work can be found in S5 Table. All the other targets for previously identified durum wheat miRNAs are reported in De Paola et al. [3]. In control and N stressed libraries, a total of 5344 unique putative target genes were identified, 4868 in Ciccio and 4443 in Svevo 2817 genes were common to all four libraries. The target genes detected only in stressed libraries amounted to 341, whereas those found only in the control material were 760 (Fig 1B).

We carried out an analysis of previously identified target genes for durum wheat conserved miRNAs [3], in order to find genes involved in N metabolism pathways. For instance, ttu-miR164d is a putative regulator of the homologue of the NAC1 protein from *Phaseolus vulgaris* (TC248477), while ttu-miR393c putatively regulates the homologue of the protein AUXIN SIGNALING F-BOX 2 from *Arabidopsis* (TC257470). Moreover, some ttu-novel miRNAs are potential regulators of genes that encode enzymes related to N pathways. For instance, the glutathione transferase F5 (TC372351) is possibly regulated by ttu-novel-74, while ttu-novel-13 and ttu-novel-61 putatively regulate genes encoding for the high-affinity nitrate transporter-activating protein 2.1 (NAR2.1) (CA641079), and for the CCAAT-box transcription factor (*CCAAT-TF*) complex WHAP6 (TC430923), respectively.

In order to evaluate a possible inverse correlation between the expression of selected miRNAs and of their corresponding predicted target genes in N stressed and in control plants, four genes targeting for differentially expressed miRNAs were chosen for qPCR analysis. In cv. Svevo, these genes were evaluated in all tissues, since in most of them the corresponding miRNAs were differentially expressed, while in cv. Ciccio, the four targets were tested only in those tissues where the regulating miRNAs were differentially expressed. The expression of target genes was normalized against RLI(a) gene, which proved to be the most stable housekeeping gene among the ones tested in our experimental conditions.

In the case of conserved durum wheat miRNAs, retained interactions with homologous target genes were observed compared to other plant species. For the novel durum wheat miRNAs, new putative target genes predicted *in silico* were taken into account. Prior to expression analyses, the target gene fragments used for qPCR experiments were sequenced to confirm the miRNA binding site and the gene sequence.

We determined the expression level of the genes encoding for a ubiquitin-conjugating E2 enzyme UBC24, PHOSPHATE 2 (*PHO2*), for the MYB3 transcription factor protein (*MYB3*, TC368630), for a protein similar to the Auxin Signaling F-box 2 of *Arabidopsis*, (*ASF2*, TC257470), and for the *CCAAT-TF* (TC430923).

Relative quantitation was performed on tissues from plants grown under N deprivation, in comparison with plants cultivated in standard conditions (2mM N).

**Fig 3:**
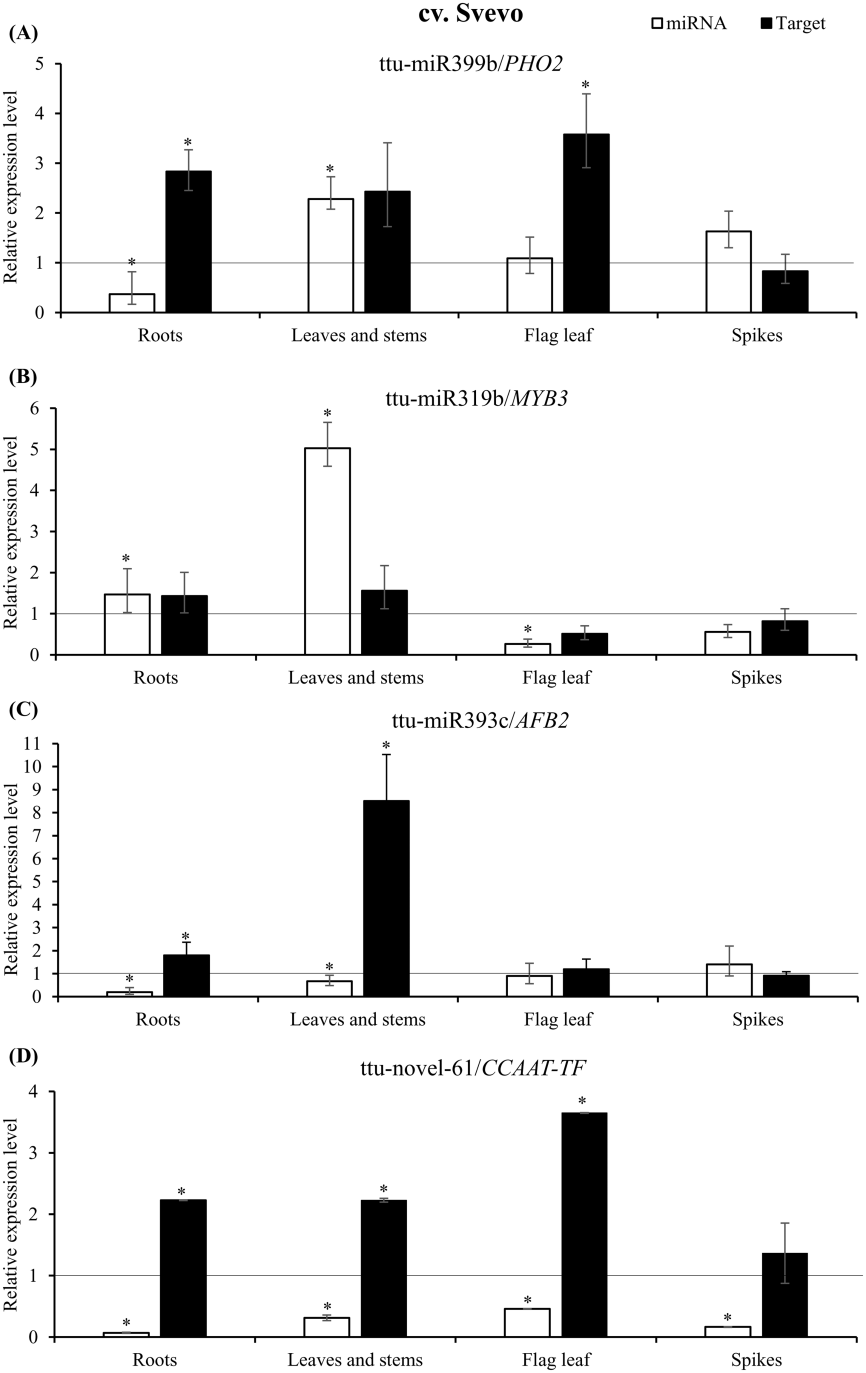
Expression analysis of putative nitrogen stress-responsive miRNAs and their target genes in Svevo cultivar. Relative expression level of selected miRNAs (white bars) and their target genes (black bars) in roots, leaves/stems, flag leaf and spikes, of Svevo cultivar grown in N stress conditions. Expression levels are calculated relative to plants grown under standard 2mM N conditions (indicated by the black line and arbitrarily set to 1). miRNA/target in this order: ttu-miR399b/*PHO2* (A), ttu-miR319b/*MYB3* (B), ttu-miR393c/*AFB2* (C), ttu-novel-61/*CCAAT-TF* (D). Target genes are indicated by abbreviations: *PHO2*, *PHOSPHATE 2*; *MYB3*, *MYB3* transcription factor; *AFB2*, auxin signalling F-box 2; *CCAAT-TF*, *CCAAT-box* transcription factor complex WHAP6. Data are means ±SE. *P* values between three N stressed biological replicates and three replicates grown under standard N conditions, were calculated using unpaired, 2-tailed student’s *t* test for each miRNA/target. Asterisks indicate statistically significant values (p-value < 0.05).

**Fig 4:**
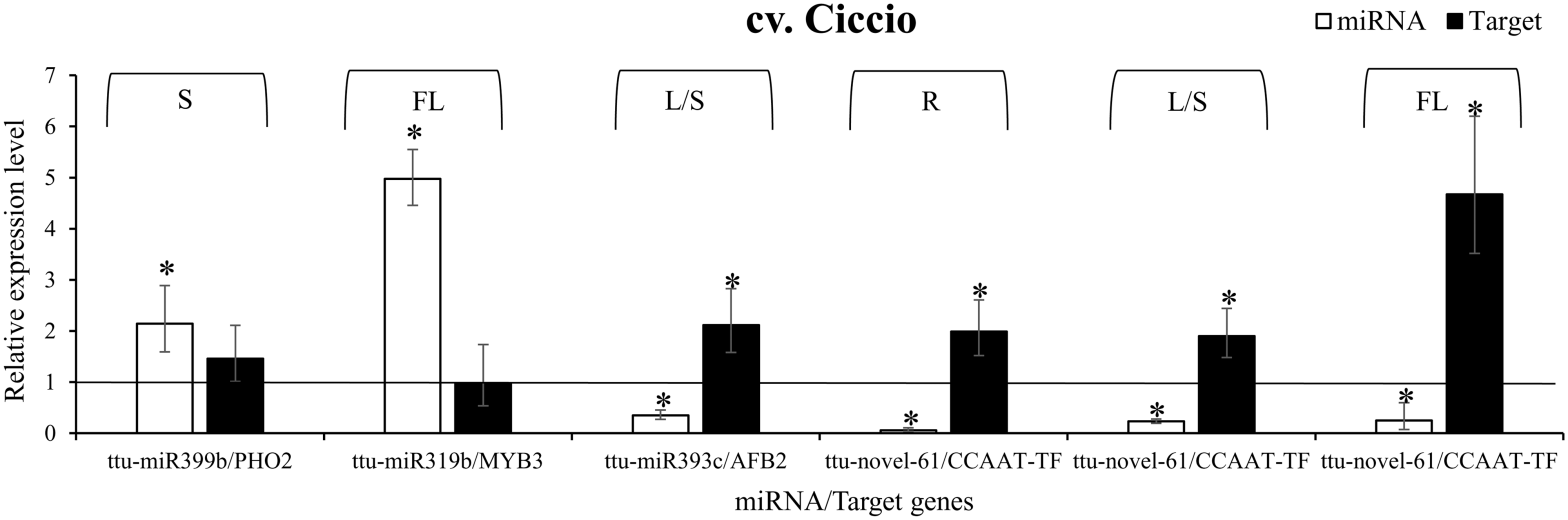
Expression analysis of putative nitrogen stress-responsive miRNAs and their target genes in Ciccio cultivar. Relative expression level of selected miRNAs (white bars) and their target genes (black bars) in roots, leaves/stems, flag leaf and spikes, of Ciccio cultivar grown in N stress conditions. Expression levels are calculated relative to plants grown under standard 2mM N conditions (indicated by the black line and arbitrarily set to 1). Tissues are indicated by abbreviations: R, roots; L/S, leaves and stems; FL, flag leaf; S, spike. Target genes are indicated by abbreviations: *PHO2*, *PHOSPHATE 2*; *MYB3*, *MYB3 transcription factor*; *AFB2*, *auxin signalling F-box 2*; *CCAAT-TF*, *CCAAT-box transcription factor complex WHAP6*. Data are means ±SE. *P* values between three N stressed biological replicates and three replicates grown under standard N conditions, were calculated using unpaired, 2-tailed student’s *t* test for each miRNA/target. Asterisks indicate statistically significant values (p-value < 0.05).

The target of miR399, *PHO2*, showed a significantly inverse correlation with ttu-miR399 in Svevo roots, being up-regulated in the plants grown in N starvation solution compared to those raised in standard conditions (Fig 3A). In Svevo leaves and stems, both *PHO2* and ttu-miR399 were up-regulated; in flag leaf the target had a higher expression level in stressed plants while ttu-miR399 was similar to the control. A slight inverse correlation for ttu-miR399/*PHO2* expression was also observed in Svevo spike, even though the fold changes were not significant (Fig 3A), while in Ciccio spike, no correlation was found (Fig 4).

MiR319b was up-regulated in roots and stems/leaves of Svevo cultivar and in flag leaf of Ciccio plants. Up-regulation of this miRNA was also observed in roots of *Zea mays* in response to transient low N condition [34]. However, although *MYB3* is described as the target gene of miR319b, in all the tissues tested and in all our experimental conditions, no inverse correlation within this couple of miRNA/target was observed, both in Svevo (Fig 3B) and in Ciccio (Fig 4).

*AFB2* expression level was compared to that of ttu-miR393c. An inverse correlation was observed in roots and leaves/stems, where a down-regulation of the miRNA corresponded to an up-regulation of its target. In flag leaf, no substantial differential expression was observed; this was also the case in the spike, where data were not statistically significant (Fig 3C).

A striking result was observed for the pair ttu-novel-61/*CCAAT-TF*, the expression of which was inversely correlated in all the tissues analyzed, both in Svevo and in Ciccio (Figs 3 and 4). In Svevo, CCAAT-box transcription factor was up-regulated especially in flag leaf (fold change 3.649) and roots (fold change 2.228), and slightly also in leaves/stems (fold change 1.677); conversely ttu-novel-61 was always down-regulated in N stress compared to standard conditions. In the spike, the same trend was observed, even though the expression of the target gene was not statistically significant (Fig 3D). Also in Ciccio, the three analyzed tissues (roots, leaves/stems and flag leaf) showed a significant down-regulation of ttu-novel-61 and up-regulation of *CCAAT-TF* (Fig 4).

**Fig 5:**
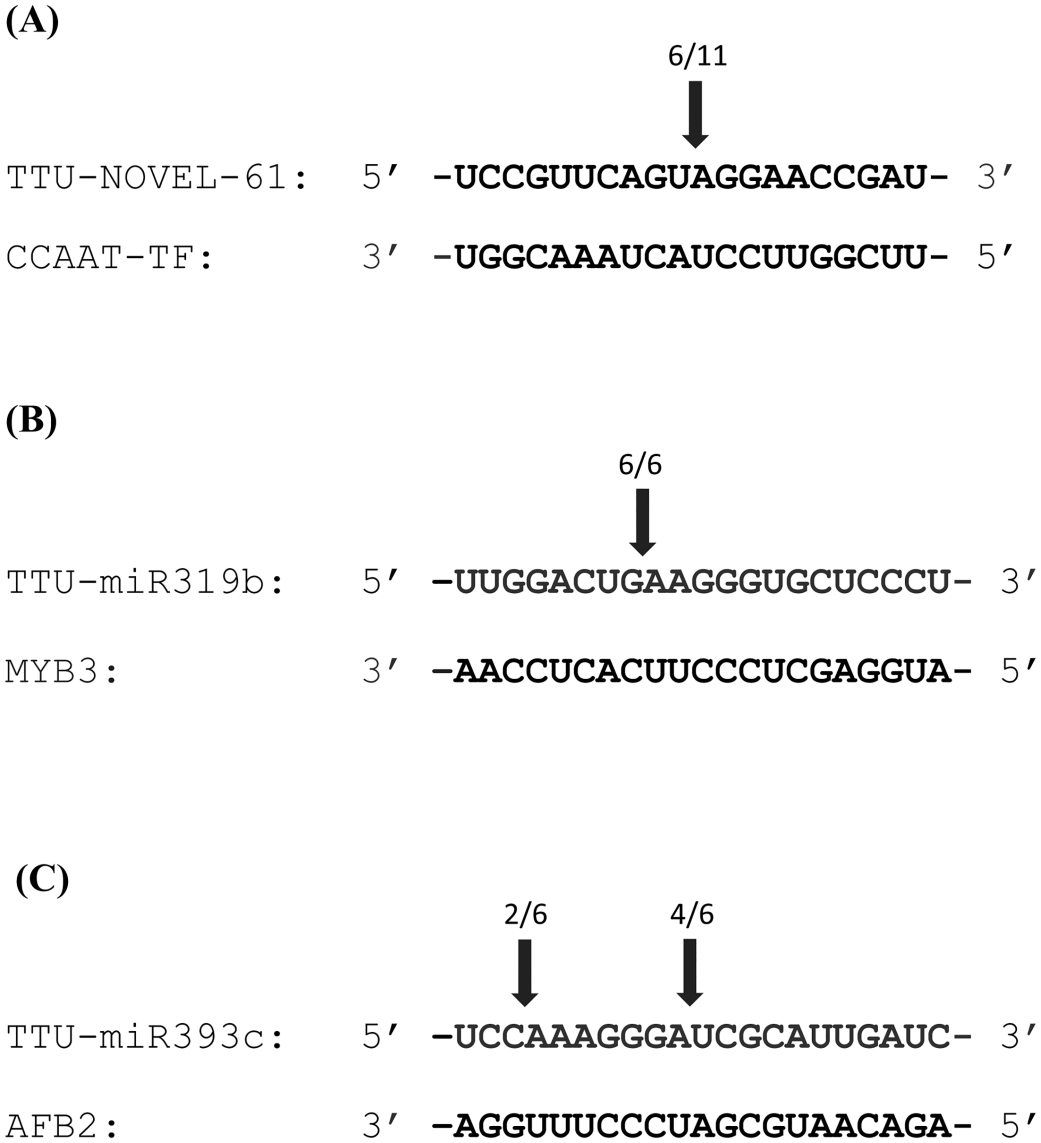
Validation of selected target genes by 5’ RACE system. Alignment between ttu-novel-61 (A), ttu-miR393c (B), and ttu-miR319b (C) and their target genes *CCAAT-TF* (Acc. No. TC430923), *AFB2 auxin signalling F-box 2* (Acc. No. TC257470) and *MYB3 transcription factor* (Acc. No. TC368630) respectively. Arrows indicate the cleavage sites. Numbers above arrows indicate number of cleaved/total clones sequenced.

To further validate *CCAAT-TF*, *AFB2*, *MYB3*, and *PHO2* target genes, we carried out a 5’ RACE analysis using gene specific primers. After cloning and sequencing 5’RACE fragments, *CCAAT-TF*, *AFB2* and *MYB3*, appeared to be cleaved as shown in Fig 5. As for *PHO2*, we did not observe a cleavage site in the expected region.

## Discussion

The identification of miRNA role in response to abiotic stress can lead to the improvement of resistance and yield in crop plants, including cereals [35,36]. Understanding the molecular changes occurring during N deprivation in plants is a fundamental step to find alternative strategies to the intensive use of N fertilizers. Various studies show the crucial role that miRNAs play in the acquisition and homeostasis of N in plants [37,38]. In this study, we used two durum wheat cultivars, which are known to have a different N use efficiency, and tested the miRNA response to N starvation conditions by evaluating various organs of the plants. Both cultivars were severely affected by N deprivation, however a higher depletion in Ciccio growth suggests that Svevo provides a better physiological response to prolonged N stress. In general, miRNA expression level under N stress could vary according to the tissue and the variety, roots and Svevo being the most responsive tissue and cultivar. Durum wheat variety-specific miRNAs were also observed in response to water stress conditions [2]. Possible target genes of durum wheat miRNAs were searched using three databases, i.e. durum wheat ESTs, bread wheat TAGI database, and also barley sequences, in order to acquire a better picture of the putative targets, since the genome of durum wheat is not yet available, whereas a high number of *T*. *aestivum* sequences can be retrieved and *H*. *vulgare* genome is well annotated. We selected some target genes based on the results obtained from miRNA expression, and, thus, the most interesting targets were evaluated in qPCR experiments, in order to assess a possible inverse correlation between the expression of a miRNA and its corresponding target.

Differences in miRNA qPCR expression between N stress and non-stress conditions were observed especially in roots, leaves/stems and flag leaf. In spikes, a lower number of miRNAs were found to be differentially expressed between N stressed and non-stressed plants in comparison to the other three tissues. Several miRNAs found differentially expressed are described to be associated with N-metabolism in other plant species. In *Arabidopsis*, miR164 is up-regulated [39] and miR167a is down-regulated [40] under N starvation conditions. MiR164 regulates the expression of NAM/ATAF/CUC (NAC) transcription factor 1 in *Arabidopsis* [39], therefore, a down-regulation of the NAC transcription factor slows down the production of lateral roots which are positively regulated by this gene. Conversely, a down-regulation of miR167 allows a higher expression of its target genes *ARF6* and *ARF8*, positively regulating the adventitious root growth [40,41]. A similar effect was observed for miR164 and miR167 in Svevo roots, suggesting that, in this durum wheat cultivar, a complex spatial miR164/miR167 regulation might also be responsible for specific root architectural adaptation to N stress. Conversely, a different response in the expression of these miRNAs was observed in Ciccio cultivar; this may derive from the fact that Svevo cultivar has a better N utilization than Ciccio [25,42].

Our results revealed a lower expression of miR827 compared to the control in the roots for Ciccio, but not for Svevo. As this miRNA is associated with phosphate absorption by targeting nitrogen limitation adaptation (NLA), its down-regulation in conditions of nitrogen starvation may be necessary to prevent over-uptake of phosphate. In *Arabidopsis* and maize, miR827 was expressed at low levels under N-starvation conditions [18,40], with a concomitant increase in the expression of its target *NLA* for *Arabidopsis* [40]. Conversely, a significant up-regulation of miR827 was observed in leaves and stems of the Svevo cultivar. A similar result was observed in the leaves of *Zea mays* under long term N-deficient, where miR827 was also up-regulated [34]. The characterization of rice osa-miR827 and its two target genes, OsSPX-MFS1 and OsSPX-MFS2, provided evidence that this miRNA may target genes with important roles in phosphate (Pi) metabolism [43].

A number of studies show a down-regulation of miR399 under N-stress conditions [33,39,43,44], and the same behavior observed in durum wheat roots in this study was also seen in the roots of ‘Shixin 828’, a largely-planted soft wheat cultivar with high nitrogen utilization [21]. PHO2 enzyme is responsible for the degradation of PHO1, a membrane-associated putative Pi transporter [45]. In phosphate starvation conditions, PHO1 expression increases, facilitating the movement of phosphate in the plant. The down-regulation of miR399 under N-stress conditions and the subsequent increase in PHO2 expression may enhance proteasome-mediated N remobilization of other putative targets such as Rubisco [37]. The crosstalk between N and P metabolism is not well known yet but there is evidence that the link between N and P is regulated by PHO2, NLA, miR399, and miR827 [46]. In this study, the negative correlation between the expression of miR399 and its target gene *PHO2* in Svevo roots might suggest an important role of this miRNA/target interaction in the adaptation of durum wheat to a low N environment.

In our study, the sequenced *PHO2* clones from 5’ RACE experiment did not show any cleavage in the expected region. In this case, translational repression might be the preferred mechanism of action for miR399b. In fact, although an almost perfect complementarity is observed between miRNA and its target sequence in plants, non-cleaving repression is a possible mechanism also in the plant kingdom [47]. Additionally, the mechanisms of cleavage and translational repression might be spatially and/or temporally separated [48].

This miR399 might be also regulated through a recently discovered mechanism called endogenous target mimicry (eTM), as observed in Arabidopsis, in order to enhance PHO2 expression [49,50].

Ttu-miR444 displayed a slight (1.489 fold change), although significant up-regulation in Ciccio roots, while it did not show significant differential expression in the other tissues and in Svevo. Recently, the soft wheat ta-miR444a was attributed a critical role in mediating plant tolerance to the N-starvation stress through modulation of the regulatory networks associated with N acquisition, cellular ROS homeostasis, and carbon assimilation [22]. The up-regulation of ta-miR444a in leaves and roots increased during N-starvation stress and regressed after *T*. *aestivum* plants were returned to standard growing conditions. In our experiment, however, durum wheat plants were grown under N deprivation during—their entire life cycle and this might have brought a different adaptive response. As it was showed in this work for N stress, other abiotic stresses also produce changes in miR444 expression in Graminaceae. For instance, in barley miR444b was down-regulated while miR444a and miR444c appear up-regulated upon boron stress conditions [51].

*MYB3* is designated as one of the target genes of miR319. However, in all of our tissues and experimental conditions tested, the expression of the miRNA and its target were not inversely correlated. MiR319 has also other potential target genes, including two *TCP* genes, homologous to the *Arabidopsis TCP2* genes that have been shown to be involved in the morphogenesis of lateral shoot organs [52] and to help control leaf senescence by regulating jasmonic acid biosynthesis [53]. Possibly, in *T*. *durum* tissues, miR319 might regulate other target genes than *MYB3*, to maintain plant metabolism balanced in N deficient conditions. In common wheat, it has been hypothesized that miR319 could improve plant tolerance to drought stress by regulating TCP transcription factors; moreover, regulation network analysis indicated that miR159 and miR319 share a number of target transcripts in wheat [54].

Experiments carried out in *Arabidopsis* showed that miR393 is transiently up-regulated in response to nitrogen treatment. Because of this miRNA up-regulation, its target Auxin-signalling F-Box 3 protein (*AFB3*) auxin-receptor is degraded and thus down-regulated [55]. miR393c was identified in the N stressed durum wheat libraries and its target gene encodes for the homolog to the *Arabidopsis* Auxin-signaling F-Box 2 (*AFB2*) [3]. Our expression data confirmed that, in Svevo roots and in leaves/stems of Svevo and Ciccio cultivars, this miRNA is down-regulated, while its putative target gene *AFB2* is significantly up-regulated. The significant miR393 down-regulation in N-absence conditions that we observed in Svevo roots might be related to the model in which a high nitrate provision would avoid further rooting by an interaction with the auxin-signalling pathway mediated by miR393/*AFB* genes. We evaluated the expression level of ttu-miR169c and ttu-novel-61. Although ttu-miR169c belongs to the miR169 family, its sequence strikingly differs from the other members of the family, since it is homologous to a miRNA located on the other arm of the precursor hairpin (bdi-miR169h-3p). Conversely, ttu-novel-61 shows a high level of sequence similarity with the standard miR169 family members. Ttu-miR169c proved to be down-regulated in N starvation conditions in all the durum wheat tissues analyzed. However, *in silico* analyses did not predict the *CCAAT-TF* factor complex WHAP6 (the standard target for miR169 members) as its target, and no other target gene was predicted for ttu-miR169c. Conversely, our *in silico* data indicate that ttu-novel-61 might regulate *CCAAT-TF*. This prediction was confirmed by qPCR analyses and the results are consistent with what has already been observed in *Arabidopsis*, where miR169 is down-regulated while its potential targets, CCAAT box-binding transcription factors (*NFYA2*, *NFYA3*, *NFYA5*, and *NFYA8*), are up-regulated [40,56]. Within the Poaceae family, expression of miR169 clearly decreases in maize N-deficient plants [18] and in bread wheat [36], where an up-regulation of *NFYA* genes is also observed. Moreover, in soft wheat, overexpression of *TaNFYA-B1* stimulates several processes such as lateral branching, up-regulation of nitrate transporters, as well as increasing N uptake and grain yields in low N conditions [36]. As such, this over-expression is an indicator of essential roles for root development and nitrogen use. In conclusion, our study showed that there is a strong down-regulation of the ttu-miR169 family members, ttu-miR169c and the ttu-novel-61, in Svevo and Ciccio plants during grain filling and under N-stress conditions. A clear negative correlation exists between ttu-novel-61 and the *CCAAT-TF* gene in most tissues of durum wheat Svevo and Ciccio cultivars. Moreover, the validity of this target was further confirmed by demonstrating the cleavage site of ttu-novel-61 in durum wheat sequence of *CCAAT-TF* WHAP6 gene.

Our findings contribute to the knowledge on durum wheat miRNAome under nitrogen stress conditions, at the grain filling stage. Although functional analyses would be necessary to confirm the role of durum wheat miRNAs and their corresponding target genes in N metabolism, the outcomes of this investigation provide important information for future studies and can be useful for crop breeding aimed at increasing durum yield, while decreasing fertilizer use, thereby contributing to the protection of the environment.

## Supporting information

S1 Table List of primers used for durum wheat miRNA retrotranscription, qPCR analyses of miRNAs and target genes, and 5' RACE assay. (XLSX)

S2 Table Phenotypic analyses on plant groups (control and stressed) of durum wheat cultivar Ciccio grown with different N concentrations. For each parameter, mean values (±standard error) and relative reductions between control and stressed conditions are presented. PH: plant height; FLA: flag leaf area; NCPP: number of culms per plant; NSPS: number of spikelets per spike; SDM: spike dry matter; FLDM: flag leaf dry matter; KNPS: kernel number per spike; KWPS: kernel weight per spike; RR: relative reduction.
(XLSX)

S3 Table List of the conserved miRNAs identified in Ciccio and Svevo durum wheat libraries from nitrogen stressed plant tissues, with at least 5 counts. Highlighted in green are known miRNAs newly identified in this work. All the other miRNAs were also detected in De Paola et al. (2016).
(XLSX)

S4 Table Novel miRNAs detected in Ciccio and Svevo durum wheat libraries from nitrogen stressed plant tissues, with at least 5 counts. (XLSX)

S5 Table List of potential targets for conserved and novel miRNAs newly identified in durum wheat Ciccio and Svevo cultivars grown under nitrogen stress conditions. (XLSX)
